# Longitudinal Changes in Anxiety and Depression Following First Implantable Cardioverter-Defibrillator Implantation: A Prospective Observational Study

**DOI:** 10.3390/medicina62101903

**Published:** 2026-10-01

**Authors:** Vincenzo Sgaramella, Antonio Gianluca Robles, Domenico Rosario Riccardo Chieppa, Domenico Gianfrancesco, Pierpaolo Vitti, Francesco Musaico, Francesca Pomarico, Paolo Pollice, Sergio Davinelli, Francesco Bartolomucci

**Affiliations:** 1 Department of Cardiology, Ospedale “L. Bonomo”, 76123 Andria, Italy; vincenzo.sgaramella89@gmail.com (V.S.); fra.pomarico@gmail.com (F.P.); paolo.pollice@yahoo.it (P.P.);; 2 Department of Medicine and Health Sciences “V. Tiberio”, University of Molise, 86100 Campobasso, Italy; 3 Department of Life, Health and Environmental Sciences, University of L’Aquila, 67100 L’Aquila, Italy

**Keywords:** implantable cardioverter-defibrillator (ICD), anxiety, depression, Hospital Anxiety and Depression Scale (HADS), sudden cardiac death, psychological distress, longitudinal study

## Abstract

*Background and Objectives*: ICD implantation is associated with psychological distress, but longitudinal patterns of anxiety and depression remain incompletely characterized. To evaluate the prevalence and longitudinal evolution of anxiety and depressive symptoms in patients undergoing first-time ICD implantation. *Materials and Methods*: This prospective study included 96 patients undergoing first ICD implantation. Anxiety and depression were assessed using the Hospital Anxiety and Depression Scale (HADS) at implantation (T0), one week post-discharge (T1), and six months later (T2). Data distribution was assessed using the Shapiro–Wilk test. Changes over time were analyzed using the Friedman test, Wilcoxon signed-rank tests, and Linear Mixed-Effects Models (LMM), with effect size (Kendall’s W) and Spearman correlations. A sensitivity analysis evaluated severe distress thresholds (HADS ≥ 11). *Results*: Significant longitudinal changes were observed for both anxiety (Friedman Q = 63.32, *p* < 0.001; Kendall’s W = 0.34) and depression (Q = 68.36, *p* < 0.001; W = 0.37). Mean HADS-A scores increased from 7.40 ± 2.70 at T0 to 9.22 ± 3.35 at T1 and 8.55 ± 2.78 at T2. Mean HADS-D scores increased from 6.53 ± 2.47 to 8.06 ± 3.02 and 8.24 ± 2.41, respectively. Clinically relevant anxiety (HADS-A ≥ 8) increased from 41.7% to 66.3% and 58.7%, while clinically relevant depression (HADS-D ≥ 8) increased from 27.1% to 54.3% and 56.5%. Sensitivity analyses for severe cases (HADS ≥ 11) confirmed consistent temporal trajectories (*p* < 0.001). Subgroup analyses (sex, indication, age) revealed no significant differences (*p* > 0.15), and no ICD shocks were delivered during follow-up. Anxiety and depression were strongly correlated at all time points (*p* < 0.001). *Conclusions*: Anxiety and depression symptoms are highly prevalent among ICD recipients and follow distinct temporal trajectories during the post-implantation course, with anxiety peaking early and depressive symptoms showing a more sustained increase in the absence of device therapies. Routine psychological screening and stage-specific psychosocial support strategies should be evaluated for integration into standard ICD care.

## 1. Introduction

Cardiovascular diseases (CVDs) remain among the leading causes of mortality and disability worldwide [1]. Patients affected by structural heart disease, inherited arrhythmia syndromes, or severe ventricular dysfunction are at increased risk of sudden cardiac death (SCD). Implantable cardioverter-defibrillators (ICDs) represent one of the most effective strategies for preventing SCD and have substantially improved survival among high-risk individuals [2].

Despite their proven clinical benefit, ICDs may have a considerable psychological impact. Patients frequently report concerns regarding potential device shocks, uncertainty about prognosis, body image alterations, and limitations in daily activities. These factors may contribute to anxiety, depression, and reduced quality of life [3,4,5,6].

Several studies have demonstrated that anxiety and depressive symptoms are common among ICD recipients. A systematic review by Magyar-Russell et al. identified substantial rates of psychological distress in this population. More recently, Ghezzi et al. confirmed the significant burden of mood symptoms and disorders among ICD recipients in a large meta-analysis involving nearly 40,000 patients [7]. The PSYCHE-ICD study further highlighted the importance of integrating psychological assessment with cardiovascular evaluation, while data from the ACQUIRE-ICD study demonstrated persistent symptoms of anxiety and depression in both primary and secondary prevention populations [8,9].

Although psychological consequences of ICD therapy have been increasingly recognized, routine mental health assessment is inconsistently implemented in clinical practice. The present study aimed to evaluate anxiety and depressive symptoms in patients undergoing first-time ICD implantation and to explore their evolution from the peri-procedural phase through six months of follow-up.

## 2. Materials and Methods

### 2.1. Study Design and Population

This prospective single-center observational study was conducted at the Intensive Care Unit–Cardiology Department of “L. Bonomo” Hospital, Andria, Italy, between August 2023 and October 2024.

Eligible participants were consecutive adult patients undergoing first-time ICD implantation for either primary or secondary prevention of sudden cardiac death, according to current European guidelines and in addition to optimized medical therapy, when appropriate [10]. All participants were required to provide informed consent and complete study questionnaires independently.

Exclusion criteria included documented past and active psychiatric disorders or ongoing psychotropic drug use, significant cognitive impairment, previous ICD implantation, prior use of a wearable cardioverter-defibrillator, and inability to complete follow-up assessments.

**Figure 1:**
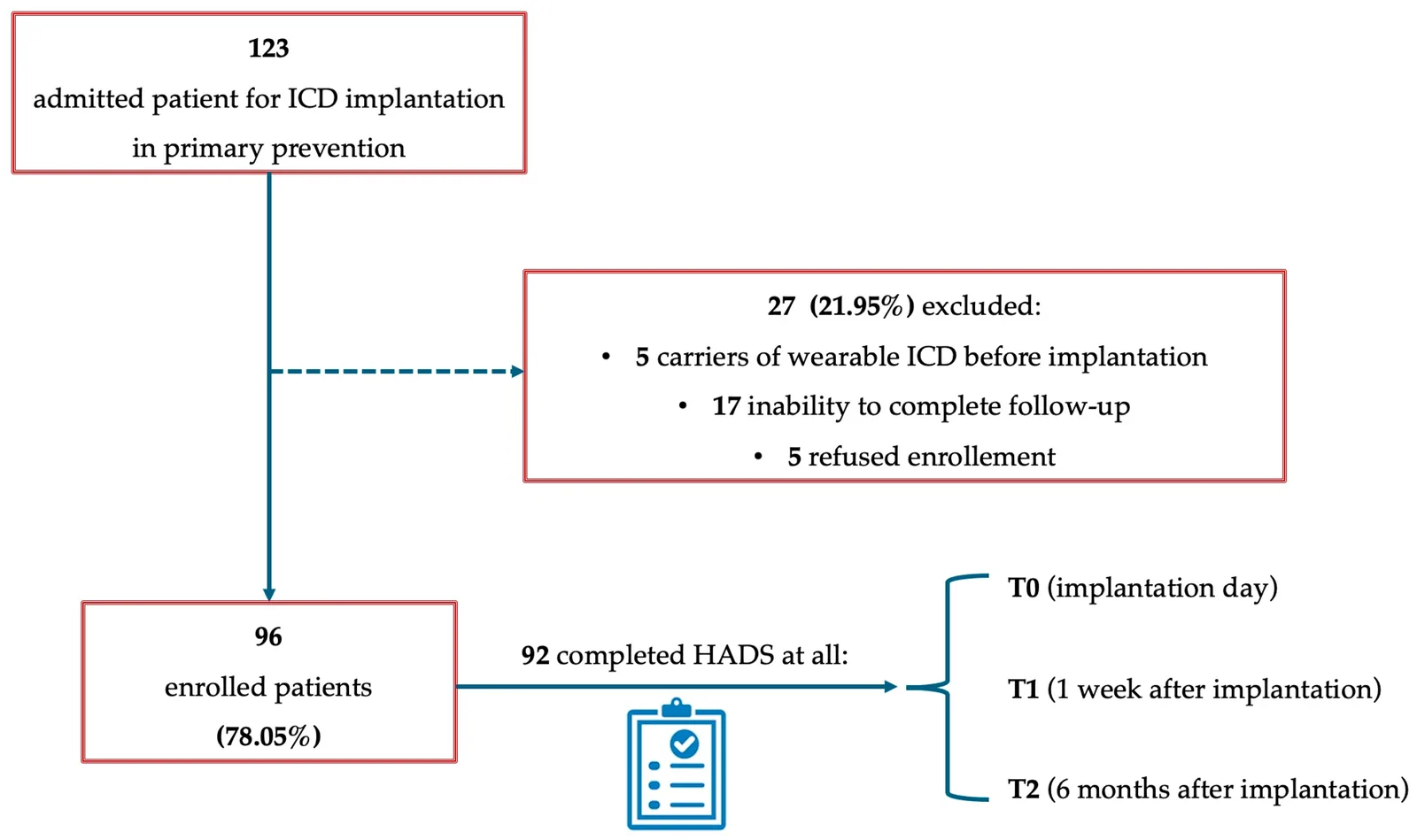
Study design flow chart.

Among 123 eligible patients, 96 were enrolled and 92 completed all scheduled evaluations. Figure 1 summarizes the study design.

### 2.2. Psychological Assessment

Psychological distress was assessed using the Hospital Anxiety and Depression Scale (HADS), a validated self-administered questionnaire consisting of 14 items divided into two subscales: anxiety (HADS-A) and depression (HADS-D) (Supplementary Materials). Each subscale ranges from 0 to 21 points, with higher scores indicating greater symptom burden. Scores ≥ 8 were considered indicative of clinically relevant symptoms.

Participants completed the HADS questionnaire at three predefined time points:

- T0: day of ICD implantation, in particular before the procedure
- T1: one week after implantation
- T2: six months after implantation

### 2.3. Clinical and Demographic Data

Demographic and clinical characteristics, including age, sex, occupational status, cardiac disease etiology, left ventricular ejection fraction, cardiovascular risk factors, medical therapies, ICD type and indication for ICD implantation (primary or secondary prevention), were extracted from medical records. All these features are summarized in Table 1.

Of note, no patients were on psychotropic drugs before enrollment or during the six-month follow-up, nor did any patient receive a diagnosis of psychiatric disease. No patients received psychological support or education as part of the standard care either before or after T0, T1 and T2 assessments. No ICD therapies (both appropriate and inappropriate) were delivered during the six months follow-up.

### 2.4. Ethical Considerations

The study was conducted in accordance with the Declaration of Helsinki and was approved by the Ethics Committee of the University of Molise (Protocol No. 23/2024, approved on 3 April 2024). The study represents the research project of V.S.’s specialization degree at the University of Molise and it was conducted at the network-affiliated Intensive Care Unit–Cardiology Department of “L. Bonomo” Hospital, Andria, Italy, as already stated.

### 2.5. Statistical Analysis

Continuous variables were expressed as mean ± standard deviation (SD) or median and interquartile range (IQR). Normality of distribution for continuous variables and score differences was evaluated using the Shapiro–Wilk test, which demonstrated non-parametric distributions for HADS scores at all time points (*p* < 0.05). Categorical variables were reported as frequencies and percentages.

An a priori sample size calculation was conducted for a repeated-measures design across three time points (T0, T1, T2). Assuming a general statistical convention for repeated-measures designs (Cohen’s f = 0.25 representing a standard medium effect size), a two-tailed significance level (α) of 0.05, and a statistical power (1 − β) of 0.80, a minimum sample size of 79 patients was required to detect longitudinal variations in psychological distress scores. To account for an estimated 10% dropout rate during follow-up, a minimum target enrollment of 88 patients was established.

Longitudinal changes in anxiety (HADS-A) and depression (HADS-D) scores across the three time points (T0, T1, T2) were primarily evaluated using the non-parametric Friedman test for repeated measures. Effect size was calculated using Kendall’s W coefficient. Post hoc pairwise comparisons between time points were performed using Wilcoxon signed-rank tests with Bonferroni correction to adjust for multiple testing (adjusted significance level: *p* < 0.017).

To confirm the statistical robustness of the primary findings and account for patient-level repeated measures and potential clinical confounders, a sensitivity analysis was performed using Linear Mixed-Effects Models (LMEM). Time point was modeled as a fixed categorical factor (with T0 as the reference level), and subject-specific random intercepts were specified to control for individual baseline variability. The LMMs were fully adjusted for relevant clinical and sociodemographic covariates, including age, sex, marital status, educational level, underlying cardiovascular etiology/diagnosis (dilated cardiomyopathy, ischemic heart disease, Brugada syndrome/channelopathies, or other structural heart disease), and ICD indication (primary vs. secondary prevention). Fixed-effect parameters were estimated using Restricted Maximum Likelihood (REML).

Associations between anxiety and depression scores at each time point were assessed using Spearman’s rank correlation coefficient. A two-sided *p*-value < 0.05 was considered statistically significant for all primary analyses. Statistical analyses were performed using IBM SPSS Statistics 29 and Python packages (statsmodels 0.15.0 and scipy.stats 1.18.1).

## 3. Results

### 3.1. Patient Characteristics

The study population consisted of 96 patients undergoing first-time ICD implantation, including 62 men (64.6%) and 34 women (35.4%). The mean age was 60.79 ± 14 years. Ischemic heart disease was the most common underlying cardiac condition. Most participants were retired at the time of enrollment. Follow-up assessments were completed by 92 patients at both T1 and T2: four patients (4.2%) were lost to follow-up due to logistical constraints unrelated to health status. Baseline comparison between completers (92) and non-completers (4) revealed no statistically significant differences in age, sex, clinical etiology, or baseline HADS scores (*p* > 0.20 for all comparisons), indicating that dropouts occurred at random.

Table 1 summarizes demographic and clinical features of the enrolled patients, as already stated before.

### 3.2. Evolution of Anxiety and Depression

A significant longitudinal variation over time was observed for both anxiety and depressive symptoms.

The Friedman test demonstrated significant changes in HADS-A (Q = 63.32, *p* < 0.001) and HADS-D scores (Q = 68.36, *p* < 0.001).

- Anxiety (HADS-A)

Mean HADS-A scores increased from 7.40 ± 2.70 at T0 to 9.22 ± 3.35 at T1, remaining elevated at 8.55 ± 2.78 at T2.

The proportion of patients with clinically relevant anxiety (HADS-A ≥ 8) increased from 41.7% (40/96) at T0 to 66.3% (61/92) at T1 and remained elevated at 58.7% (54/92) at T2.

- Depression (HADS-D)

Mean HADS-D scores increased from 6.53 ± 2.47 at T0 to 8.06 ± 3.02 at T1, and 8.24 ± 2.41 at T2.

Clinically relevant depressive symptoms (HADS-D ≥ 8) were present in 27.1% (26/96) at baseline, increasing to 54.3% (50/92) at T1 and 56.5% (52/92) at T2.

### 3.3. Linear Mixed-Effects Models for Longitudinal Outcomes

The Linear Mixed-Effects Models fully corroborated these longitudinal trends, showing that the fixed effect of time remained highly significant for both anxiety (β_T1_ = 1.94, *p* < 0.001; β_T2_ = 1.57, *p* < 0.001) and depression (β_T1_ = 1.57, *p* < 0.001; β_T2_ = 1.73, *p* < 0.001), even after adjusting for all clinical and sociodemographic covariates (age, sex, education, marital status, cardiac disease etiology, and ICD indication).

### 3.4. Effect Size and Post Hoc Analysis

Effect size analysis demonstrated a moderate effect for anxiety (Kendall’s W = 0.34) and a moderate-to-large effect for depression (Kendall’s W = 0.37).

Moreover, the post hoc analysis showed:

- Anxiety increased from T0 to T1 (*p* < 0.001) and remained higher at T2 vs. T0 (*p* < 0.01); no significant difference between T1 and T2.
- Depression increased from T0 to T1 (*p* < 0.001) and from T0 to T2 (*p* < 0.001); no difference between T1 and T2.

### 3.5. Correlation Between Anxiety and Depression

A strong positive correlation was observed at all time points:

- T0: r = 0.693, *p* < 0.001
- T1: r = 0.680, *p* < 0.001
- T2: r = 0.703, *p* < 0.001

**Figure 2:**
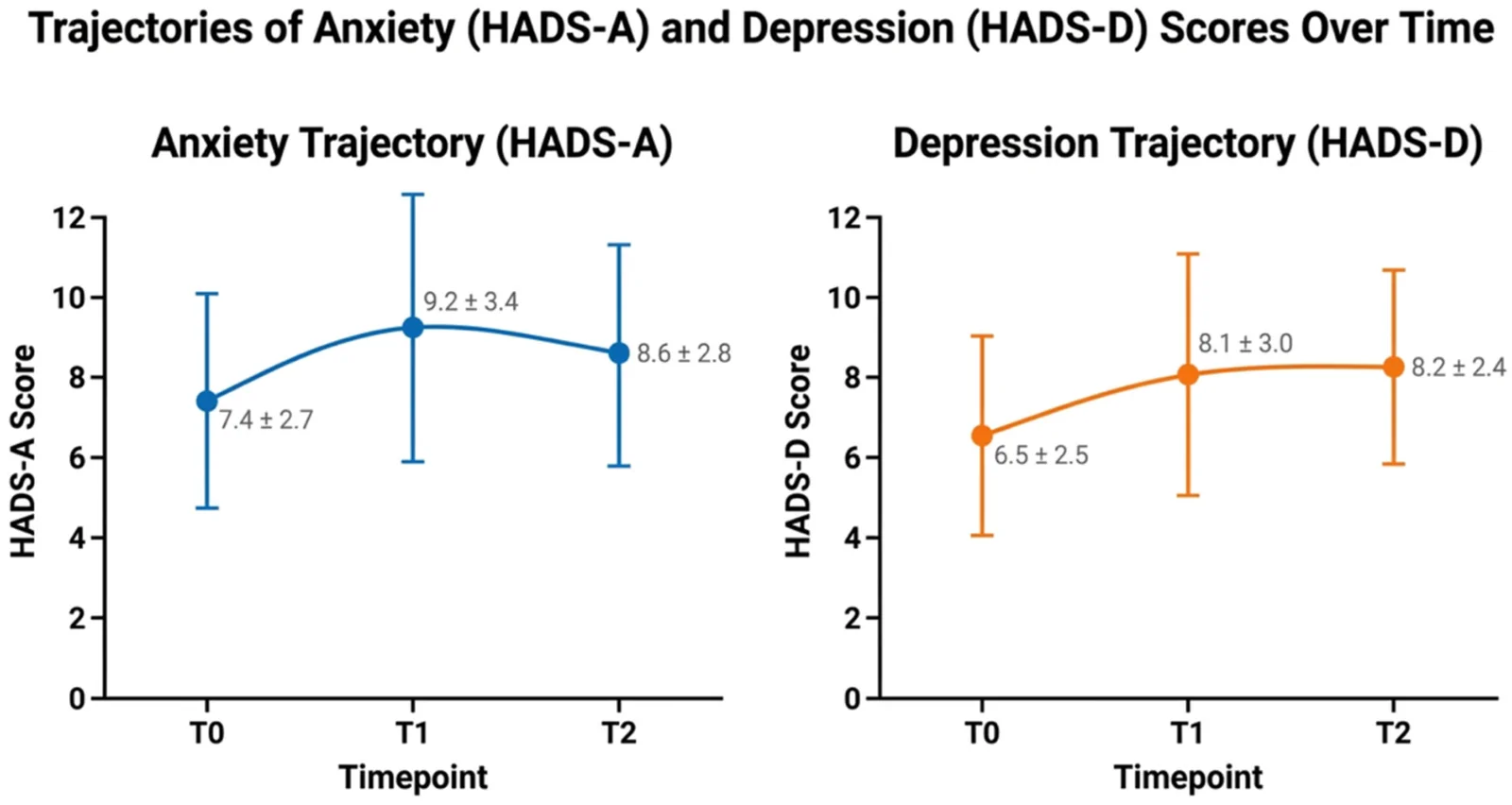
Longitudinal changes in anxiety (HADS-A) and depression (HADS-D) scores. Anxiety increased significantly at 1 week after ICD implantation and remained elevated at 6 months. Conversely, depressive symptoms showed a progressive increase over time following ICD implantation. Values are expressed as mean ± SD.

**Figure 3:**
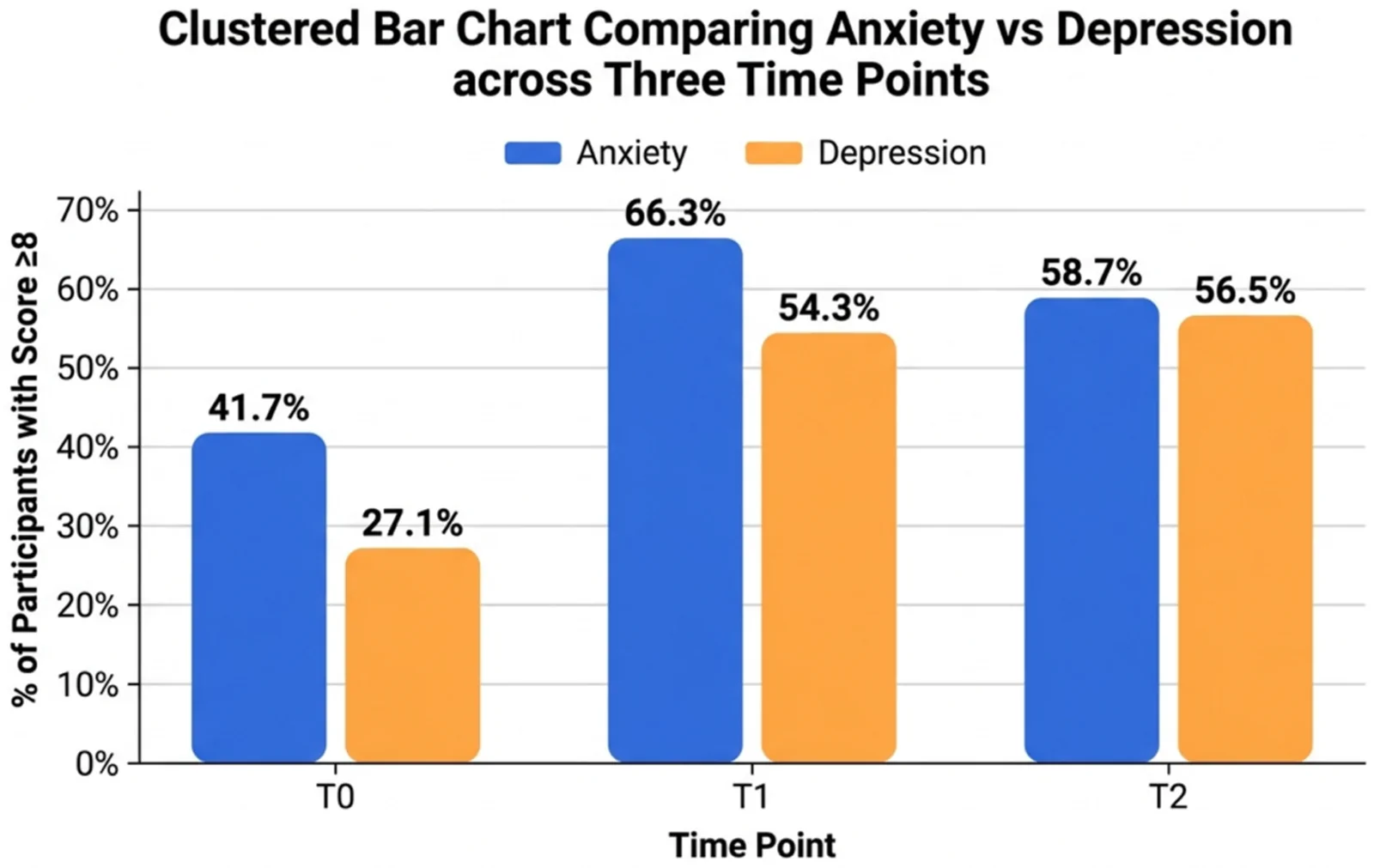
Prevalence of clinically relevant anxiety and depression (HADS ≥ 8). Both anxiety and depression increased after ICD implantation and remained elevated during follow-up.

**Figure 4:**
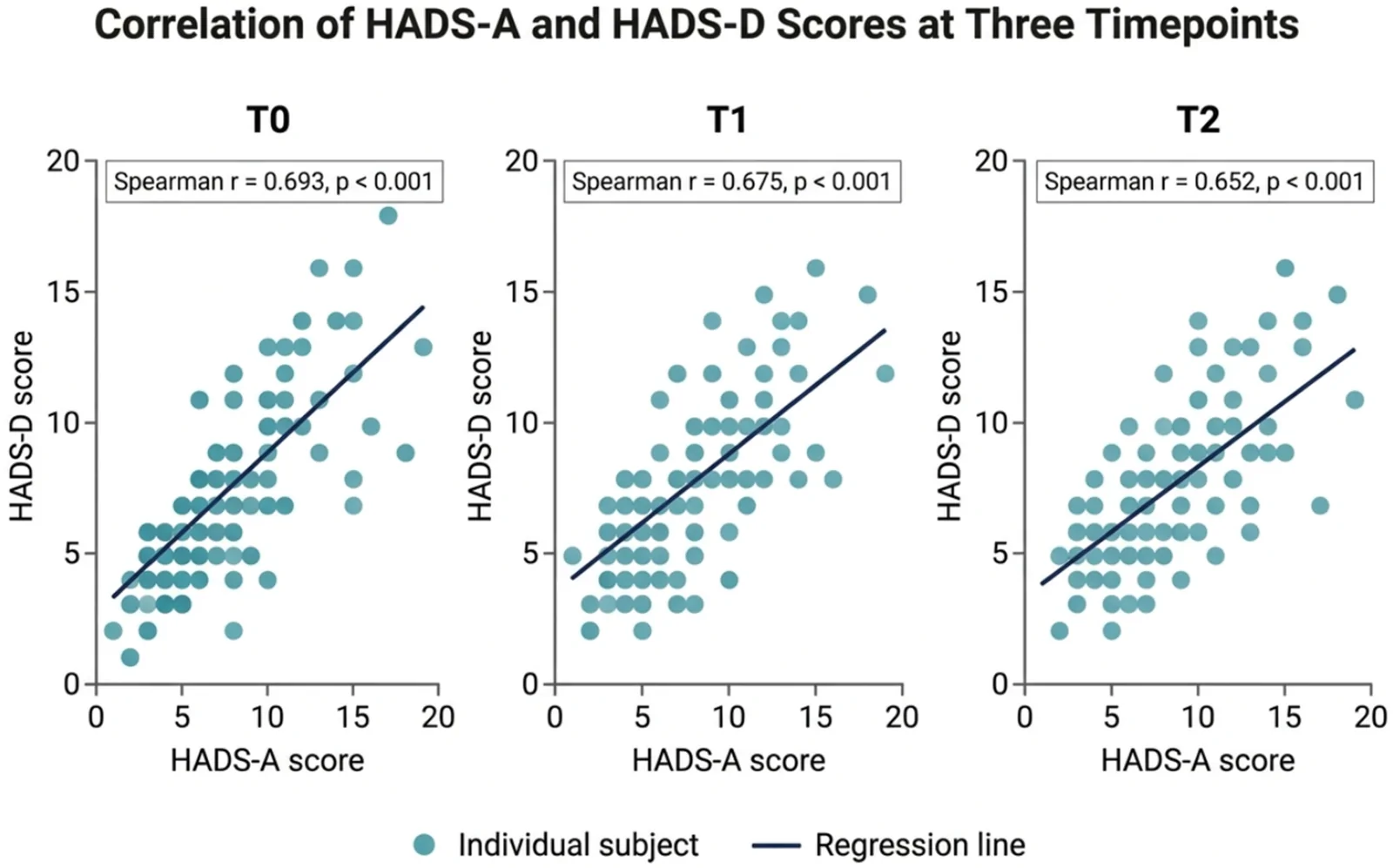
Correlation between anxiety and depressive symptoms during follow-up. Scatter plots showing the relationship between HADS-Anxiety (HADS-A) and HADS-Depression (HADS-D) scores at baseline (T0), one week after implantation (T1), and six months after implantation (T2). A strong positive correlation was observed at all assessment points (T0: r = 0.693; T1: r = 0.680; T2: r = 0.703; *p* < 0.001 for all comparisons).

Of note, all the above-mentioned results are reported in Figure 2, Figure 3 and Figure 4.

### 3.6. Subgroup Analysis and Sensitivity Testing

No statistically significant differences in longitudinal HADS-A or HADS-D trajectories were observed according to sex (Mann–Whitney U = 982.5, *p* = 0.41), indication for ICD implantation (primary vs. secondary prevention; U = 1012.0, *p* = 0.58), age groups (<65 vs. ≥65 years; *p* = 0.34), or educational level (*p* = 0.29).

Sensitivity analyses using a stricter threshold for probable clinical cases (HADS ≥ 11) demonstrated consistent temporal patterns. Severe anxiety (HADS-A ≥ 11) increased from 12.5% (12/96) at T0 to 33.7% (31/92) at T1 and 25.0% (23/92) at T2 (*p* < 0.001). Severe depression (HADS-D ≥ 11) increased from 5.2% (5/96) at T0 to 20.7% (19/92) at T1 and 18.5% (17/92) at T2 (*p* < 0.001).

## 4. Discussion

### 4.1. Main Findings

This prospective study confirms that anxiety and depressive symptoms are common among patients undergoing first-time ICD implantation and may persist beyond the immediate peri-procedural period. While the association between ICD therapy and psychological distress has been extensively documented [3,4,5,6], our findings contribute additional longitudinal evidence by characterizing the distinct temporal evolution of anxiety and depression from implantation through six months of follow-up.

It is worth noting that baseline (T_0_) HADS scores were non-zero and slightly elevated. Assessing baseline parameters on the day of implantation naturally reflects acute pre-procedural stress, hospital admission anxiety, and recent cardiac events rather than a pre-morbid steady state. On the one hand, extensive literature reports a high prevalence of depressive and anxiety symptoms in patients with cardiovascular disease, with clinically relevant symptoms affecting up to 20–30% of this population prior to major interventions [5,11,12]. On the other hand, these elevated scores most likely capture acute emotional reactivity to the immediate stress of the underlying cardiac event, the impending device implantation, and the specific clinical pathway (primary vs. secondary prevention). While baseline elevations may partially stem from underlying subclinical or undiagnosed mood symptoms, they may primarily represent a heightened baseline state triggered by a high-stress medical scenario. Ultimately, this T_0_ assessment serves as a meaningful clinical benchmark upon which the subsequent evolution of post-implantation psychological symptoms is superimposed.

An interesting finding of the present study is the different trajectories observed for anxiety and depressive symptoms over time. The highest mean anxiety scores were recorded during the first week following implantation, suggesting that procedural stress, adaptation to the device, concerns regarding future ICD therapies, and uncertainty about lifestyle changes may contribute to increased psychological distress in the early post-implantation phase. Although anxiety scores slightly decreased at six months, they remained above baseline values, indicating incomplete psychological recovery.

In contrast, depressive symptoms demonstrated a more gradual and sustained increase throughout follow-up. Both mean HADS-D scores and the proportion of patients exceeding clinically relevant thresholds remained elevated at six months. This divergent pattern suggests that anxiety and depression may reflect different phases of psychological adaptation to ICD therapy. Anxiety appears to be predominantly related to the immediate emotional response to device implantation, whereas depressive symptoms may emerge or consolidate during the longer-term adjustment process. These findings support the concept that psychological adaptation to ICD therapy extends well beyond hospitalization and requires continued monitoring during follow-up.

The magnitude of these changes was confirmed by effect size analysis, which demonstrated a moderate effect for anxiety (Kendall’s W = 0.34) and a moderate-to-large effect for depression (Kendall’s W = 0.37), supporting the clinical relevance of the observed psychological trajectories. This pattern was also reflected in the dispersion of scores, with greater variability observed at T1 followed by a partial reduction at T2, suggesting a heterogeneous psychological response in the early post-implantation phase and a subsequent, although incomplete, adaptive process over time. Post hoc analyses further clarified temporal patterns, indicating that anxiety increases primarily occur in the early post-implantation phase, whereas depressive symptoms show a more persistent increase over time. The divergent temporal trajectories—anxiety peaking acutely at 1 week (T1) and depression climbing progressively through 6 months (T2)—suggest a sequential psychological reaction where acute anxiety may act as a vulnerability factor for subsequent depressive adaptation. While a formal cross-lagged panel analysis would provide valuable mechanistic insights, we felt it exceeded the sample size capacity and scope of the current dataset. To this end, cross-lagged modeling is advisable in in future larger multicenter cohorts.

Another noteworthy observation concerns the prevalence of clinically relevant depressive symptoms. Although anxiety has traditionally received greater attention in ICD recipients, the proportion of patients with HADS-D scores ≥ 8 more than doubled during follow-up, increasing from 27.1% at baseline to 56.5% at six months. By the end of follow-up, depressive symptoms were nearly as prevalent as anxiety symptoms, suggesting that depression may represent an underrecognized component of the psychological burden associated with ICD therapy. Furthermore, self-reported depressive symptoms in cardiovascular cohorts must be interpreted cautiously, as somatic and affective manifestations may significantly overlap with chronic heart failure symptoms or subtle cognitive impairment [13]. Nevertheless, these findings reinforce the need for systematic screening strategies that assess both anxiety and depression rather than focusing predominantly on fear-related symptoms.

Our results are consistent with previous literature. Magyar-Russell et al. reported substantial prevalence rates of anxiety and depression among ICD recipients, while the recent meta-analysis by Ghezzi et al. confirmed the significant burden of mood disorders in this population [5,7]. Similarly, findings from the PSYCHE-ICD study emphasized the close interaction between psychological and cardiovascular health, and data from ACQUIRE-ICD reported persistent symptoms across both primary and secondary prevention groups [8,9]. However, unlike many previous studies that relied on cross-sectional assessments or longer follow-up intervals, the present study provides prospective real-world data capturing both the early and intermediate phases following first ICD implantation. This design allowed a more detailed description of the temporal evolution of psychological distress in routine clinical practice.

Importantly, we observed a strong correlation between anxiety and depression symptoms at all time points. Whether they only frequently coexist or represent interconnected components of psychological distress in ICD recipients is an open question deserving further evaluation. Nevertheless, their coexistence may identify a subgroup of patients at greater risk of impaired quality of life and poorer adaptation to device therapy, warranting a comprehensive mental health assessment and subsequent interventions, if indicated.

No significant differences were observed according to sex or indication for ICD implantation. These findings are in agreement with previous reports suggesting that psychological distress may affect a broad spectrum of ICD recipients irrespective of implantation indication, emphasizing the need for universal rather than selective psychological surveillance strategies [3,4,5]. Notably, no ICD therapies (both appropriate and inappropriate) were delivered during follow-up, and no psychotropic drugs were prescribed. This indicates that the observed early surge in anxiety and sustained depression was not driven by shock delivery, but rather by procedural stress, body image adaptation, and anticipatory anxiety regarding potential future therapies.

Overall, the novelty of our findings does not lie in demonstrating the existence of anxiety and depression among ICD recipients, which is already well established, but rather in the prospective characterization of their distinct temporal patterns and persistence during the first six months following implantation. These observations may help clinicians identify critical periods for psychological assessment and develop stage-specific interventions aimed at improving both emotional well-being and overall patient-centered outcomes.

### 4.2. Clinical Implications and Future Perspectives

From a clinical perspective, the present findings strongly support the integration of routine psychological screening into standard ICD care pathways, particularly during the early post-implantation phase. Stage-specific interventions could be established: early post-discharge (Week 1), with immediate screening and anxiety management focused on procedural stress, wound care, and fear of shocks; and medium-term (6 Months), with screening for insidious depressive symptoms and routine integration into cardiac rehabilitation.

Simple, validated instruments such as the HADS could easily be implemented in clinical practice to facilitate the prompt identification of high-risk patients requiring targeted support. Furthermore, emerging evidence suggests that structured psychological interventions, digital support platforms, structured patient education, and resilience-based programs may significantly attenuate psychosocial burden and foster long-term adaptation [14,15,16].

Another crucial dimension involves patient education and shared decision-making. Active patient engagement, a clear understanding of device functioning, and participation in treatment choices have been shown to substantially improve psychological adjustment following ICD implantation [17,18]. Although these factors were not directly evaluated in our cohort, they represent vital targets for future longitudinal research. Optimizing communication strategies and pre-implantation counseling may identify modifiable targets capable of mitigating emotional distress and enhancing quality of life in first-time ICD recipients.

Future longitudinal studies employing Item Response Theory (IRT) or symptom-network analyses could further clarify whether specific sub-dimensions or individual symptoms (e.g., specific autonomic anxiety features or anhedonic symptoms) are disproportionately sensitive to early post-implantation distress.

### 4.3. Methodological Considerations and Statistical Robustness

A major strength of the present study lies in its rigorous longitudinal design and statistical methodology. To avoid the common pitfall of underpowered observational studies, sample size was determined a priori. Furthermore, while non-parametric repeated-measures analyses (Friedman test) confirmed significant overall psychological trajectories, we implemented Linear Mixed-Effects Models (LMM) as a robust sensitivity analysis. This advanced longitudinal modeling approach allowed us to account for subject-level baseline heterogeneity (via random intercepts) and to control for a broad panel of potential clinical and sociodemographic confounders—including age, sex, educational background, marital status, ICD indication (primary vs. secondary prevention), and underlying cardiac etiology. Crucially, the LMEM fully corroborated our primary findings, suggesting that the post-implantation surges in anxiety and depression are robust independent phenomena. This dual analytical framework provides high statistical confidence, demonstrating that post-implantation psychological changes are robust phenomena associated with the peri-procedural and early adaptation period.

## 5. Limitations

Several limitations of the present study should be acknowledged.

First, this was a single-center study, which may restrict the generalizability of our findings to broader populations. Although the a priori sample size calculation relied on standard medium effect size assumptions (Cohen’s f = 0.25) rather than population-specific preliminary estimates, given the paucity of published prospective data on HADS trajectories at early post-implantation intervals, it confirmed that the sample size provided robust statistical power to detect significant longitudinal changes. Furthermore, while the 6-month follow-up effectively captured acute and medium-term psychological responses post-implantation, longer follow-up periods are needed to evaluate long-term psychological adaptation to the ICD.

Second, psychological assessment relied exclusively on the self-administered HADS questionnaire. Although HADS is a widely validated instrument in cardiovascular research, structured psychiatric interviews could provide a more comprehensive clinical evaluation of emotional distress.

Third, variables related to health literacy, patient education, the quality of informed consent, and shared decision-making were not systematically evaluated, despite their potential influence on psychological outcomes.

Fourth, while our longitudinal analyses were adjusted for major sociodemographic and clinical confounders using Linear Mixed-Effects Models, certain clinical parameters—such as longitudinal changes in NYHA functional class and optimization of medical therapy—were not routinely tracked and could indirectly impact emotional burden.

Finally, the observational design without a non-ICD control group (e.g., cardiac patients with similar clinical features not receiving an implantable device) precludes isolating the psychological impact of ICD implantation from the burden of the underlying cardiovascular disease itself. Nevertheless, establishing such a control group was beyond the primary scope of this study, which aimed specifically to describe the prevalence and temporal evolution of distress in first-time ICD recipients.

## 6. Conclusions

This prospective study suggests that anxiety and depressive symptoms are common among patients undergoing first-time ICD implantation and change significantly during the first six months following the procedure. Anxiety showed an early increase after implantation, whereas depressive symptoms exhibited a more gradual and persistent rise over time.

The persistence of clinically relevant symptoms suggests psychological distress and highlights the importance of considering mental health as an integral component of ICD care. Notably, depressive symptoms became increasingly prevalent during follow-up, suggesting that they may represent an underrecognized aspect of the burden associated with ICD therapy.

The main contribution of this study is the prospective characterization of the distinct evolution of anxiety and depression symptoms after first ICD implantation. Routine psychological screening, patient education, shared decision-making, and targeted psychosocial support should be considered to improve patient-centered outcomes. Further multicenter studies are needed to confirm these findings and optimize psychological care strategies for ICD recipients.

## Figures and Tables

**Table 1:** Patient Demographic and Clinical Characteristics.

Category	
Age	60.79 (±14.00)
LVEF	41.77 (±11.17)
Female	34 (35.4%)
Education:	
Primary School	44 (45.9%)
Middle School	12 (12.3%)
High School	30 (31.8%)
University	10 (9.9%)
Occupation:	
Retired	52 (54.2%)
Marital Status:	
Married	72 (75%)
CV risk factors:	
Obesity	58 (60.4%)
dyslipidemia	38 (39.6%)
Hypertension	58 (60.4%)
Diabetes	58 (60.4%)
Cardiac Disease Etiology:	
Ischemic Heart Disease	38 (39.6%)
Dilated Cardiomyopathy	20 (20.8%)
Channelopathies (e.g., Brugada)	18 (18.8%)
Other Structural Cardiomyopathies	20 (20.8%)
Type of prevention:	
Primary	47 (49%)
Secondary	49 (51%)
Type of implanted ICD:	
Single chamber	30 (31.3%)
Dual chamber	48 (50%)
CRT-D	10 (10.4%)
S-ICD	8 (8.3%)
Medical therapy:	
βB	58 (60.4%)
ACEi/ARB/ARNi	78 (81.2%)
SGLT2i	78 (81.2%)
Diuretics	78 (81.2%)
Lipid-lowering therapy	38 (39.6%)
Antidiabetic drugs (other than SGLT2i)	58 (60.4%)
AADs (other than βB)	49 (51%)
ICD interventions during follow-up (appropriate and inappropriate)	0 (0%)

Continuous variables are expressed as mean (±SD). Discrete variables are expressed as N * (%). * Total enrolled patients: 96. LVEF: left ventricular ejection fraction; CV: cardiovascular; ICD: implantable cardioverter defibrillator; CRT: cardiac resynchronization therapy; S-ICD: subcutaneous ICD; βB: beta-blockers; ACEi: angiotensin-converting enzyme inhibitors; ARB: angiotensin receptor blockers; ARNI: Angiotensin Receptor-Neprilysin Inhibitor; SGLT2i: sodium-glucose cotransporter-2 inhibitors.

## Data Availability

Data is contained within the article or Supplementary Materials.

## Supplementary Materials

The following supporting information can be downloaded at: https://www.mdpi.com/article/10.3390/medicina62101903/s1, Attachment S1. Hospital Anxiety and Depression Scale (HADS).
